# Research on Traditional Chinese Medicine: Domain Knowledge Graph Completion and Quality Evaluation

**DOI:** 10.2196/55090

**Published:** 2024-08-02

**Authors:** Chang Liu, Zhan Li, Jianmin Li, Yiqian Qu, Ying Chang, Qing Han, Lingyong Cao, Shuyuan Lin

**Affiliations:** 1 School of Basic Medical Sciences Zhejiang Chinese Medical University Hangzhou China; 2 Breast Disease Specialist Hospital of Guangdong Provincial Hospital of Chinese Medicine Guangdong Provincial Hospital of Chinese Medicine Guangzhou China; 3 Zhejiang Chinese Medical University and Gancao Doctor Chinese Medicine Artificial Intelligence Joint Engineering Center Zhejiang Chinese Medical University Hangzhou China; 4 School of Medical Technology and Information Engineering Zhejiang Chinese Medical University Hangzhou China

**Keywords:** graph completion, traditional Chinese medicine, graph quality evaluation, graph representation, knowledge graph

## Abstract

**Background:**

Knowledge graphs (KGs) can integrate domain knowledge into a traditional Chinese medicine (TCM) intelligent syndrome differentiation model. However, the quality of current KGs in the TCM domain varies greatly, related to the lack of knowledge graph completion (KGC) and evaluation methods.

**Objective:**

This study aims to investigate KGC and evaluation methods tailored for TCM domain knowledge.

**Methods:**

In the KGC phase, according to the characteristics of TCM domain knowledge, we proposed a 3-step “entity-ontology-path” completion approach. This approach uses path reasoning, ontology rule reasoning, and association rules. In the KGC quality evaluation phase, we proposed a 3-dimensional evaluation framework that encompasses completeness, accuracy, and usability, using quantitative metrics such as complex network analysis, ontology reasoning, and graph representation. Furthermore, we compared the impact of different graph representation models on KG usability.

**Results:**

In the KGC phase, 52, 107, 27, and 479 triples were added by outlier analysis, rule-based reasoning, association rules, and path-based reasoning, respectively. In addition, rule-based reasoning identified 14 contradictory triples. In the KGC quality evaluation phase, in terms of completeness, KG had higher density and lower sparsity after completion, and there were no contradictory rules within the KG. In terms of accuracy, KG after completion was more consistent with prior knowledge. In terms of usability, the mean reciprocal ranking, mean rank, and hit rate of the first N tail entities predicted by the model (Hits@N) of the TransE, RotatE, DistMult, and ComplEx graph representation models all showed improvement after KGC. Among them, the RotatE model achieved the best representation.

**Conclusions:**

The 3-step completion approach can effectively improve the completeness, accuracy, and availability of KGs, and the 3-dimensional evaluation framework can be used for comprehensive KGC evaluation. In the TCM field, the RotatE model performed better at KG representation.

## Introduction

### Background

Traditional Chinese medicine (TCM) has unique advantages for diagnosing and treating a variety of diseases [1]. It also played a remarkable role in preventing and treating COVID-19 during the global pandemic [2]. The prerequisite to the effectiveness of TCM relies on accurate syndrome differentiation and treatment determination. However, a manual syndrome differentiation process results in subjective differences [3]. Applying artificial intelligence technology to auxiliary diagnosis and treatment will contribute to standardization in this area [4]. A review has suggested that the accuracy of TCM intelligent syndrome differentiation models has reached the application standard [5]. However, deep learning models that perform well often suffer from a lack of explainability, and they are heavily reliant on data, which limits their application. Knowledge graphs (KGs) can integrate domain knowledge into intelligent models, reduce data dependency, and enhance explainability [6]. Therefore, many studies have constructed KGs in the TCM field. However, the variable quality of existing KGs [7] impacts their ability to effectively represent knowledge and support tasks such as intelligent diagnosis, question answering, and prescription recommendation. Given that the technology for constructing KGs is becoming increasingly pervasive, we contend that the absence of a comprehensive knowledge graph completion (KGC) and quality evaluation system tailored to TCM is a critical factor contributing to this variation.

Research on KGC and quality evaluation is essential in the field of TCM. First, the existing construction work of TCM KGs predominantly relies on a single knowledge source and lacks a methodology for exploring rules from different knowledge sources. Second, there is a scarcity of methods to identify abnormal connections within KGs. KGC necessitates a foundation of accurate knowledge. However, semiautomatic KG construction may incorporate contradictory, erroneous, or incomplete knowledge [8], which needs to be discovered and corrected. Currently, the TCM field KG lacks a systematic approach for identifying inaccurate knowledge and rectifying errors. Third, the majority of existing methods for graph completion evaluation focus solely on specific algorithm evaluation metrics, lacking a stereoscopic evaluation framework that assesses the overall quality of the completed graph.

There are 2 current challenges. First, theories and methods for KGC are scarce within the TCM field, which makes it difficult to complete knowledge at different levels and identify inaccurate knowledge in the graph. Second, there is a lack of a KGC quality management system and evaluation criteria that are specifically tailored for TCM domain knowledge.

### Goal of This Study

To address these challenges, we designed a completion plan based on the characteristics of TCM domain knowledge. This plan targets the 3 levels of knowledge—explicit, implicit, and tacit—and systematically completes the KG from the perspectives of path, ontology, and entity. We also proposed a completion evaluation system that includes 3 dimensions: completeness, accuracy, usability. For each of these dimensions, we developed specific evaluation metrics.

The contributions of this paper are summarized here. First, based on the characteristics of TCM knowledge, a 3-step completion plan consisting of “path-ontology-entity” was proposed. This plan not only identifies and corrects inaccurate knowledge but also enhances the completeness of the graph, providing a methodological reference for related research in the field. Second, under the KG quality management framework, we proposed a quality evaluation system for TCM KGC, providing a reference for comprehensive and multidimensional KG evaluation and promoting KG quality improvement in the field.

### Related Work

#### Application and Development of KG in TCM

The application of KG technology in the field of TCM dates back over 20 years. The Traditional Chinese Medicine Language System (TCMLS) defines the most basic semantic types and semantic relationships in the field of TCM [9]. General Formal Ontology (GFO)-TCM is a mid-level ontology that is built upon the foundation of TCMLS using a top-down approach [10]. Based on modern literature or by integrating multiple literature sources, researchers have constructed KGs in various subdomains of TCM, including syndromes [11], medical cases [12,13], prescriptions and herbs [14], and health preservation [15], among others. KG has been applied in the TCM field in information retrieval, question answering [16-18], visual analysis [19], auxiliary diagnosis [20], and treatment, among others. However, there are still shortcomings in the explication of the syndrome differentiation process, the fusion of ancient and modern knowledge, and the combination of theory and clinical practice [7]. The study of KG should effectively address practical problems in TCM clinical practice and integrate the characteristics of the TCM knowledge system [21]. Therefore, this study focused on the reports of KG in TCM auxiliary diagnosis and treatment. Sun et al [22] built a TCM auxiliary diagnosis and treatment system for rheumatoid arthritis, which was based on the knowledge from TCM classics, providing doctors with guidance on diagnosis and treatment knowledge. Fu et al [23] constructed a KG of acute abdominal pain using Neo4j, used a diagnosis and treatment reasoning algorithm based on association rule mining combined with random walk, and provided information services and technical support for primary doctors by recommending personalized diagnosis and treatment plans for cases.

In current research, the intelligent syndrome differentiation in TCM is frequently represented as a classification problem, where deep learning models receive symptom information as input and output syndrome categories. Graph-based representation learning can provide domain knowledge to the model. For instance, Li et al [24] transformed 20,000 medical records into medical record graphs and used them as inputs to graph convolutional networks to learn graph embedding of prostate cancer features. This approach effectively maps the features of prostate cancer and facilitates the diagnostic process. Li et al [25] embedded knowledge regarding cerebral palsy from KGs into tensors and integrated them into recurrent neural networks, achieving a diagnostic accuracy of 79.31%. Subsequent fine-tuning with electronic medical records elevated the model’s accuracy to 83.12% [25].

#### Overview of KGC Methods in the Medical Field

KGC is an application of knowledge reasoning. Knowledge reasoning is essential for addressing the incompleteness, potential biases, and errors found in KGs, as well as for inferring hidden information between knowledge entities [26]. Methods for KGC can be broadly categorized into 3 types: rule-based, vector-based, and neural network-based.

Rule-based reasoning [27] has the advantage of using prior knowledge to provide accurate and traceable reasoning with high explainability. However, the downside is the difficulty in enumerating all the rules and the limited generalization ability. Rule-based reasoning includes predicate logic rules and ontology rules.

Vector-based reasoning methods first project entities and relations into a vector space. Triplets serve as input to learn vector representations through constraint functions. Predicted triplets are generated by fixing the head entity and applying a representation model. By converting reasoning problems into vector calculation problems, vector-based reasoning is more efficient and easier to train than traditional reasoning approaches. Common graph representation learning models include TransE [28], TransH [29], TransR [30], TransD [31], RotatE [32], RESCAL [33], DistMult [34], and ComplEx [35]. However, this approach primarily focuses on direct relations between entities and overlooks indirect paths among entities in graphs. In addition, it lacks explainability. Moreover, embedding methods degrade with increasing sparsity and unreliability of the KG [36].

Neural network–based reasoning involves learning entity features and semantic sequences from prior knowledge. It then uses neural networks to identify the linkage path between 2 entities to aid in reasoning by predicting the relation path. This approach has the advantage of using the graph structure and hidden node information to the fullest extent possible. However, it also has drawbacks, including high model complexity, large data requirements, and poor explainability.

#### Current Status of KG Quality Assessment

Xue and Zou [37] summarized the current research on KG quality management and proposed 5 dimensions for KG evaluation: accuracy, consistency, completeness, timeliness, and redundancy. The methods for KG quality evaluation can be categorized into 4 types: human-based, statistical-based, rule-based, and comprehensive. We reviewed the literature related to the completion and quality assessment of medical KGs published over the past 5 years and classified the evaluation methods into the following 3 dimensions, with reference to the definition by Xue and Zou [37]: (1) completeness, (2) accuracy, (3) usability.

For completeness, a scale was designed, and medical experts were invited to manually evaluate the KG data authority and data volume [38]. For accuracy, using mean average precision, an evaluation index for target detection and classification, the prediction triplet was evaluated as a classification problem [39]. Weighted sampling was conducted on the completed (predicted) triples. Experts judged the correctness of these triples, and the accuracy was calculated [40]. Through complex network analysis methods such as clustering [41] and t-distributed stochastic neighbor embedding visualization [42] (a visualization method for data after dimensionality reduction), KG disease classification knowledge was summarized and compared with prior knowledge, aiming to determine whether the data distribution of the constructed graph was consistent with prior knowledge. For usability, KG quality was evaluated using the effectiveness of graph representation. For instance, KG can be vectorized through graph representation algorithms to predict tail entities based on head entities and relationships. This is frequently used to compare the completion effects between different algorithms. Common metrics are the mean rank (MR) of the correct answer, the reciprocal rank of the first correct answer (mean reciprocal ranking [MRR]), and the normalized discount cumulative return of the first N predicted tail entities (Hits@N) [43]. Additionally, there are studies that have evaluated KGs by examining their performance on downstream tasks, such as the introduction of area under the receiver operating characteristic curve indicators in drug reuse and target identification [44].

Among the aforementioned methods and indicators, the direct manual evaluation of KG is affected by subjective factors and was not used in this study. The mean average precision metric is appropriate for predicting multicategory triples, which is not consist with the design of our study protocol. The purpose of this study was to design a general evaluation method for KGC in the field of TCM. Since indicators such as the receiver operating characteristic curve require specific downstream tasks for their application, the intermediate stage of KG utilization—KG representation—was chosen as the criterion for usability evaluation. With the remaining methods as reference, we designed a TCM KGC evaluation system (detailed in the Quality Evaluation section). To quantitatively assess the quality of the KG, we introduced some metrics derived from complex network analysis.

#### Introduction of the TCM Knowledge System

In TCM theory, *syndrome* (Zheng Hou) refers to the classification and summary of relatively stable symptoms and signs during disease occurrence and development. *Syndrome* is the diagnostic conclusion in TCM. For the sake of convenience, we will refer to the symptoms and signs that patients have as *symptoms* in the following sections. The cognitive process of determining the syndrome is referred to as *syndrome differentiation* (Bian Zheng). This process involves inferring the *pathogenesis factors* (Bing Ji, including disease location, disease nature, and disease state) from the symptoms and then composing the symptoms based on specific combinations and weights of the pathogenesis factors [45]. The process of determining the treatment plan is called *prescription determination* (Lun Zhi), which involves formulating the main prescription based on the syndrome and adjusting the medication according to the symptoms.

**Figure 1:**
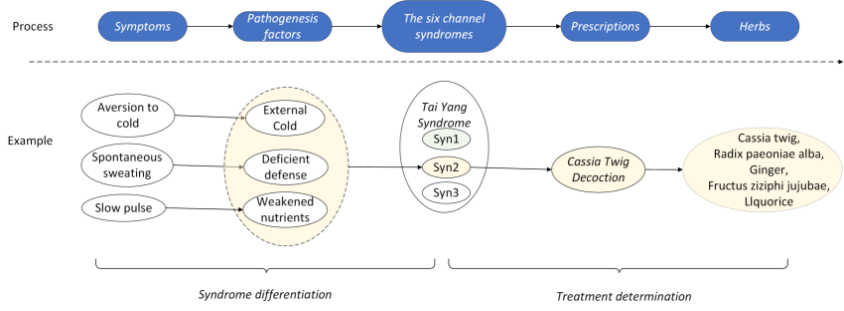
The Six Channel Syndrome Differentiation process.

Among many syndrome differentiation methods, “The Six Channel Syndrome Differentiation” is one that categorizes disease syndromes into 6 major categories: *Tai Yang, Yang Ming, Shao Yang, Tai Yin, Shao Yin*, and *Jue Yin*. It further subdivides each syndrome level under these major categories. For instance, *Tai Yang* includes 3 syndromes: *Tai Yang Shang Han*, *Tai Yang Zhong Feng*, and *Feng Han Liang Gan*. When a patient presents with *symptoms* such as aversion to cold, spontaneous sweating, and slow pulse, which indicate pathogenesis factors of external cold, deficient defense, and inadequate nutrients, the syndrome can be identified as *Tai Yang Zhong Feng,* and the main prescription would be *Cassia Twig Decoction* (Gui Zhi Tang; shown in Figure 1).

Referring to research in cognitive science on brain cognition and memory, human knowledge can be divided into 3 levels according to the different types of performance: explicit knowledge, implicit knowledge, and tacit knowledge [46]. Its definition and embodiment in the TCM field are as follows: Explicit knowledge refers to the knowledge already present in the text, some of which can be derived from the first-order predicate logic of the original and others through the reasoning of multistep connections. Examples of this include the law of syndrome differentiation and the treatment rules in ancient medical books. Implicit knowledge refers to knowledge that is not present in the text but exists in the domain scheme defined by the ontology. For example, the symptoms and contraindications that are not documented in ancient books can be inferred from explicit knowledge. Tacit knowledge is not explicitly stated in the text and can be uncovered through data mining methods, for example, the clinical manifestations of comorbidities and syndromes that are present in electronic medical records.

The KGC plan in the TCM field should be designed based on the characteristics of knowledge at these 3 levels.

#### Discussion of the KGC Method in the TCM Field

The medical field requires knowledge that is accurate, rigorous, and traceable; hence, KGC should be interpretable. This requirement influences our selection of completion methods. Although graph representation learning is relatively popular, it was used solely as an evaluation method in this study due to its limited explainability.

Path inference can fully use the paths between nodes for rule mining. The classic path ranking algorithm (PRA) learns KG relation characteristics through random walks and can predict potential relationships between 2 entities using the path between them [47]. Path-based reasoning has good performance and explainability [39]. Liu [48] optimized the link prediction model based on the PRA, which was applied for TCM KGC of famous prescriptions. Shao et al [49] constructed KGs for famous TCM doctor experiences with diagnosing and treating lung cancer, used the RED-GNN model, and mined implicit knowledge using relational path reasoning. Outlier detection can be considered a special form of path reasoning that can automatically identify abnormal connections in the KG. This study introduced outlier detection to improve KG accuracy.

Data mining methods have been widely used to discover hidden rules in TCM [50]. Association rule mining, a prevalent data mining method in TCM, explores the relation between item sets in data sets. It is frequently applied to mine relations between symptoms and syndromes, medications and syndromes, and symptoms and medications [51]. The Apriori algorithm is a Boolean, single-dimensional, single-layer association rule that links and prunes all item sets generated by multiple scans, leveraging the Apriori property to improve mining efficiency [52]. During the process of syndrome differentiation, core information is derived from knowledge associated with symptoms, pathogenesis factors, and TCM syndromes. The relation between *symptoms* and *pathogenesis factors* is relatively consistent. Consequently, the primary focus of KGC is to mine rules between *symptoms* and *syndromes*. Additionally, the (prescription-treat-symptom) triples can provide supplementary information to the *symptoms* vector in knowledge graph embedding (KGE). Therefore, KGC should primarily focus on completing the aforementioned 2 types of relations. Although these relations may be frequently absent or irregularly distributed in ancient literature, they are readily available in clinical case data. For instance, ancient literature lacks records of tongue and pulse manifestations for diabetes, which is also known in TCM as *Xiao Ke*.

In summary, path reasoning is suitable for reasoning explicit knowledge, ontology-based reasoning is suitable for mining implicit knowledge, and data mining is suitable for discovering tacit knowledge.

## Methods

### Ethical Considerations

This study was approved by the Ethics Committee of The Second Affiliated Hospital of Zhejiang Chinese Medical University (approval number: 2022051-01).

### TCM KGC Methodology

Based on the characteristics of TCM knowledge and targeting the 3-level knowledge system of “explicit knowledge, implicit knowledge, and tacit knowledge,” this study constructed a 3-order completion plan of “path-ontology-entity.”

The first order completes explicit knowledge at the “path” level. It focuses on the unique structure of the path in the KG and mines knowledge using multistep predicate logic based on path reasoning.

The second order completes the “ontology” level of implicit knowledge. It uses ontology-based rule reasoning to make implicit framed knowledge explicit by generating new triples.

The third order completes tacit knowledge at the “entity” level. It uses data mining methods to identify unestablished associations between entities. This paper used association rule mining, a widely used data mining method, as an example.

### Proposed Method

#### Task Description

**Figure 2:**
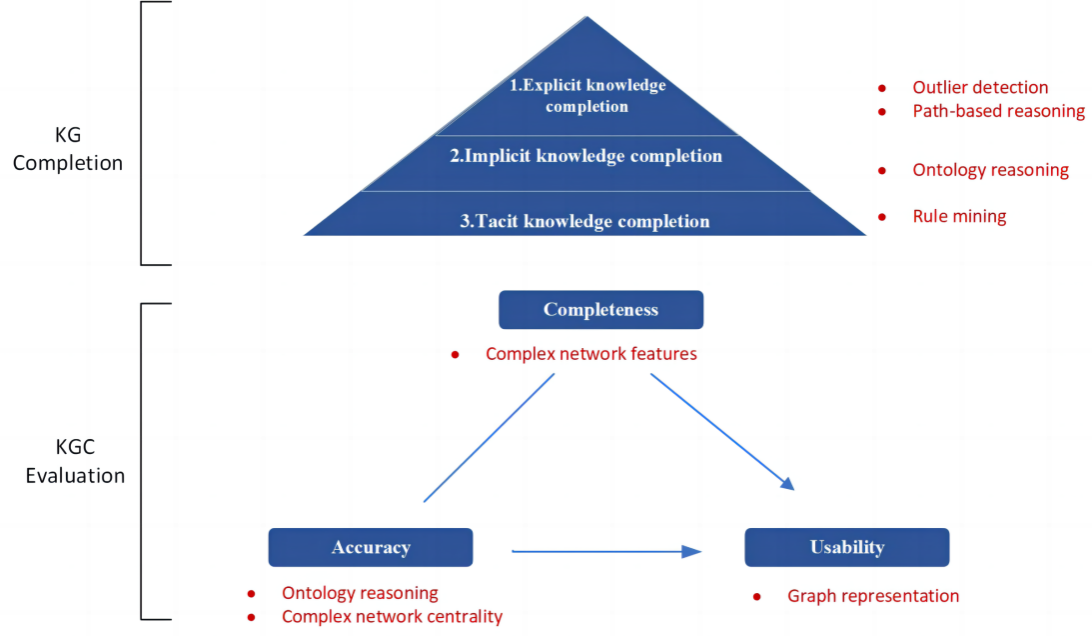
Methodology of the research. KG: knowledge graph; KGC: knowledge graph completion.

We focused on the completion and evaluation of TCM domain KGs, aiming to achieve a completion that improves both accuracy and completeness and to comprehensively evaluate the quality of the graph after completion. Specifically, the task includes the following steps: (1) KGC and (2) KGC evaluation. In KGC, (1) explicit knowledge completion involves identifying the isolated triples in the recognition graph by detecting outliers and mine (Syndrome-Manifest-Symptom) and (Prescription-Treat-Symptom) rules from the clinical case data set using path-based reasoning; (2) implicit knowledge completion involves using ontology-based deductive reasoning and the discovery of contradictory knowledge to supplement missing knowledge and correct inaccuracies; and (3) tacit knowledge completion involves using association rules to mine (Syndrome-Manifest - Symptom) knowledge in KG. These generated triples are incorporated into the graph. KGC evaluation encompasses the following 3 dimensions: completeness, accuracy, and usability. Metrics specific to each dimension are used to compare the graph before and after completion, thereby assessing the quality of completion. Specifically, completeness evaluation is grounded in statistical methods, which characterize the graph by complex network features. Accuracy evaluation uses a multifaceted approach, incorporating ontology reasoning and complex network centrality analysis. Usability evaluation is based on a statistical method that compares the impact of KGC with that of KGE. The methodology is shown in Figure 2.

#### KGC

##### Explicit Knowledge Completion

Regarding outlier detection, outliers are data points that deviate from the norm of the data set and are deemed inconsistent with the rest of the data. In our study, outliers were defined as isolated subgraphs that lacked interconnections with other subgraphs. These isolated subgraphs were manually reviewed and categorized, and ontology reasoning was used to complete the relations among subgraphs that provided valuable diagnostic information, thereby integrating them with other subgraphs.

Regarding relation prediction based on path inference, the PRA was used to mine potential relations involving the antecedents of *syndrome* or *prescription* and the consequent of *symptom*. The results were ranked based on the number of supporting paths. Feature extraction involved generating paths using a random walk approach and selecting the feature set of paths. Feature calculation entailed computing the feature value P(s→t;π_j_) for each training sample, which represents the probability of transitioning from node s to node t through relationship path π_j_. A classifier was trained using the feature values of the training samples and used to infer the existence of a target relationship between two entities. The score function is shown in equation 1:

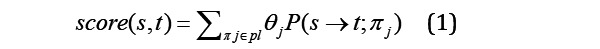



Rules with more than 2 supporting paths were selected. The predicted results were further filtered using ontology inference based on the triples of (Symptom-Correspond to-Pathogenesis Factors) and (Prescription-Treat-Syndrome). The *symptom* in the rules was converted to *pathogenesis factor*, and the *prescription* was converted to *curable syndrome*. Only the rules that were consistent with the triples of (Syndrome-Contains-Pathogenesis Factors) in the KG were selected. The antecedent and consequent of the rules were used as the head and tail entities of the predicted triples, respectively. These entities were connected by relation to obtain 2 types of predicted triples: (Syndrome-Manifest-Symptom) and (Prescription-Treat-Symptom).

The accuracy, recall, and *F*_1_-score of the predicted triples were calculated using back-to-back annotations from 2 experts as the standard. The predicted triples from both methods were merged into the KG for completion.


Precision=TP/(TP+FP) **(2)**
Recall=TP/(TP+FN)
*F*_1_=(2P·R)/(P+R)

##### Implicit Knowledge Completion

For ontology reasoning, based on the description logic of the ontology, the triples within the KG were completed and corrected. The relation properties, mainly involving transitivity, symmetry, and mutual exclusivity, were defined as shown in Table 1. Triples featuring transitive and symmetric relations were reasoned, and the deduced triples were integrated into the graph. Contradictory triples were detected through mutual exclusivity and corrected upon expert review.

**Table 1:** Definition of relations' properties in ontology.

Property	Definition	Relation
Transitivity	Relation P to ∀ entity x, y, z: P (x, y) and P (y, z) includes P (x, y).	Contain (for relation “clinical manifestation is”)
Symmetry	Relation P to ∀ entity x, y: P (x, y) is equal to P (y, z).	Differential diagnosis is
Mutual exclusivity	Entity x, y simultaneously exists with P (x, y) and R (x, y), but a contradiction arises between relations P and R.	Treat and contraindication is

##### Tacit Knowledge Completion

For relationship prediction based on association rule mining, the Apriori algorithm was used to mine rules in medical records, with *syndrome* as the antecedent and *symptom* as the consequent. The reliability of the rules was evaluated by the value of lift, where a lift greater than 1 indicates a positive correlation between the 2 items. The support of item set X is defined as the proportion of transactions in the data set that contains the item set. The confidence of a rule is defined as confidence (X≥Y), which can be interpreted as an estimate of the probability P(Y|X) [53]. The lift measure for a rule (X≥Y) is calculated as shown in equation 3:

lift(X≥Y) = confidence(X≥Y)/support(Y) = P(Y|X)/P(Y) **(3)**

The parameters for the Apriori algorithm were set to a minimum support of 10%. The resulting rules were sorted by lift value, and rules with a lift greater than 1 were considered as the mining results for the model.

#### Quality Evaluation

##### Overview of the Dimensions

In this study, we proposed a KGC evaluation system tailored for the TCM domain KG, which consists of the following 3 dimensions: (1) completeness, which assesses whether the graph includes relevant data of interest in the domain; (2) accuracy, which measures the graph’s reflection of facts, ensuring consistency with prior knowledge; and (3) usability, which evaluates the difference in KGE before and after graph completion. Since the data quality dimension is abstract, specific measurement criteria were defined in this study to apply and quantify these dimensions in practice.

##### Completeness

The overall structural completeness of the graph before and after completion was evaluated through topological properties. The specific indicators included the number of nodes, relations, degree, degree distribution (maximum degree, average degree), and network density. The degree of a node, denoted as *k*, was defined as the number of edges directly connected to a node. The average degree of all nodes in the network was denoted as *<k>*. The density *ρ* of a network with *N* nodes was defined as the ratio of the actual number of edges *M* to the maximum possible number of edges.

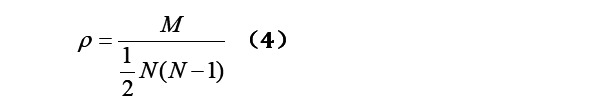



Network density can measure the sparsity of a network. A network density approaching zero indicates that the actual number of edges in the network is of lower order than *N^2^*; thus, the network is considered sparse.

##### Accuracy

Step 1 integrates rule-based and human-based approaches: We used mutual exclusion rules in ontology reasoning to assess the alterations in contradictory knowledge before and after completion (the method used was similar to that described in the Explicit Knowledge Completion subsection in the Methods section).

Step 2 uses a statistical method–based approach: complex network centrality. We analyzed the distribution of symptoms and prescriptions before and after completion and compared them with prior knowledge. The specific indicators and their meanings were identified as described in the following paragraphs.

Closeness centrality (CC) quantifies how close a node is to the center of the network. By calculating the CC for prescription nodes, we were able to identify the central prescriptions in the graph and compared them against prior knowledge. In this study, central prescriptions as defined in prior knowledge were those primary and secondary prescriptions for treating syndromes listed in the *Treatise on Febrile Diseases* textbook [54]. The CC calculation method for node *i* is as described in equation 5:

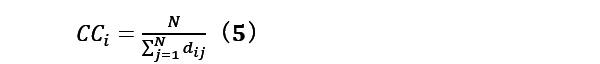



where *dij* represents the distance from node *i* to vertex *j* and N represents the number of nodes in the network.

The k-core value refers to the maximum subgraph of a graph in which each node has a degree of at least k and no more nodes can be added without reducing any node’s degree below k. By using the k-core value of symptom nodes, we were able to pinpoint the symptom groups emphasized in the diagnostic system within the KG.

##### Usability

The effectiveness of graph completion was assessed through KG representation. We used all triples in the KG as samples and randomly divided them into a training set and validation set in a 7:3 ratio. Various representation models were used to represent entities and relations. Negative samples were generated by replacing the tail entity of the actual triples with a randomly selected entity. Both positive and negative samples were fed into the model. During the training process, each example was assigned a loss function (see Table 2) to ensure that the score discrepancy between positive and negative samples exceeded the predefined margin, facilitating feedback for model updating. After each epoch of model training, the validation set was used to predict model performance. The L2 norm was adopted to measure the distance between the head entity’s mapping vector and the tail entity, resulting in the predicted score for each entity when acting as a tail. By sorting all triples according to the predicted score of the tail entity, the relative ranking of true triples among all triples can be obtained.

**Table 2:** Loss function of knowledge graph representation models.

Model	Score function
TransE	–‖*h*+*r*–*t*‖
DistMult	‹*h*,*r*,*t*›
ComplEx	Re(‹*h*+*r*–*t*›)
RotatE	–‖*h*•*r*–*t*‖

We compared the MR, MRR, and Hits@N before and after completion using the same model:

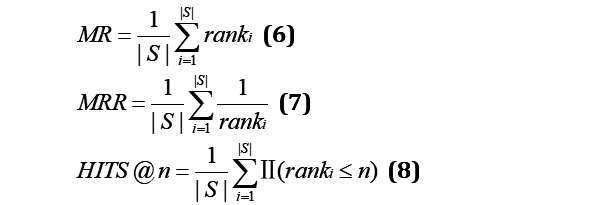



where S is the set of triples, |S| is the number of triple sets, rank_i_ is the prediction rank of the ith triple, and Ⅱ is the indicator function (1 if the condition is true, 0 otherwise).

## Results

### Data Sources

**Figure 3:**
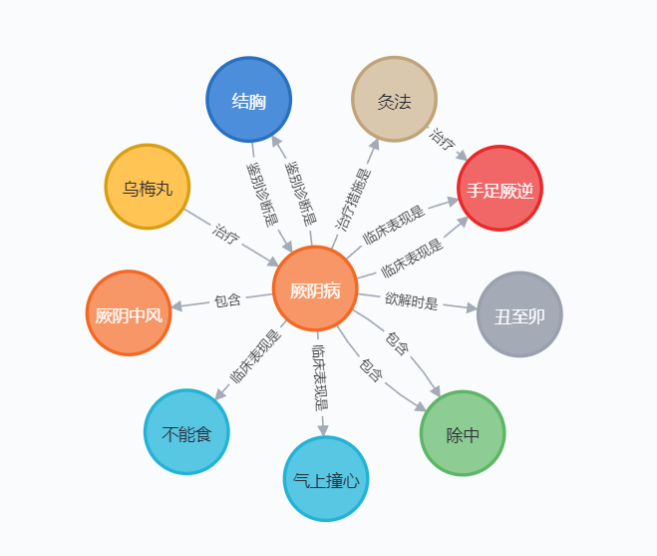
Part of a knowledge graph based on “Treatise on Febrile and Miscellaneous Diseases.” Node names: 厥阴病: Jue yin syndrome; 厥阴中风: Jue yin Zhong Feng; 乌梅丸: Fructus Mume Pill; 结胸: Chest Binding; 灸法: moxibustion; 手足厥逆: extremely cold limbs; 丑至卯: 1 to 5 am; 除中: Chu Zhong; 不能食: no appetite; 气上撞心: a feeling of gas rushing up toward the thorax. Relation names: 临床表现是: the clinical manifestation is; 包含: include; 治疗: treat; 鉴别诊断是: differential diagnosis is; 治疗措施是: the treatment method is; 欲解时是: time for recovery.

In our previous work, we developed a KG based on the ancient medical text *Treatise on Febrile and Miscellaneous Diseases.* We standardized and systematically organized the core concepts and their relations, particularly the *Six Channel Syndromes*, pathogenesis factors, and symptoms, through data mining and literature research. The graph comprised 1255 nodes and 4519 edges. Nodes and edges related to *Jue Yin* syndrome are shown in Figure 3. Additionally, we standardized the medical records of 470 clinical cases treated with classical prescriptions for rule mining in KGC.

### KGC Results

#### Explicit Knowledge Completion

##### Outliers

A total of 9 isolated subgraphs were identified, of which 6 were related to “Fangzheng” (as shown in Table 3). Fangzheng belongs to the subsyndromes under the secondary syndrome, which specifically refers to the syndrome treated by a certain prescription and is mainly used to represent knowledge related to differential diagnosis in the KG. By supplementing the (Prescription-Treat-Fangzheng) triple, connections between Fangzheng and other subgraphs can be established. A total of 52 triples were added. The remaining 3 isolated subgraphs were related to treatment methods and seldom-used prescriptions, which were not completed.

**Table 3:** List of outliers.

Isolated subgraphs	Nodes of subgraphs	Related to
1	(Li Zhong Pill Syndrome)理中丸证 (Red Halloysitum Rubrum and Limonitum Decoction Syndrome)赤石脂禹余粮汤证 (Meretrix Powder Syndrome)文蛤散证 (Poria-Liquorice Decoction Syndrome)茯苓甘草汤证, (Wu Ling Powder Syndrome)五苓散证 (Xie Xin Decoction Syndrome)泻心汤证 (Sanwubai Powder Syndrome)三物白散证	Fangzheng
2	(Capejasmine and Fermented Soybean Decoction Syndrome)栀子豉汤证 (Capejasmine-Ginger-Fermented Soybean Decoction Syndrome)栀子生姜豉汤证 (Capejasmine-Liquorice-Fermented Soybean Decoction Syndrome)栀子甘草豉汤证	Fangzheng
3	(Cassia Twif and Radix Aconiti Lateralis Preparata Decoction Syndrome)桂枝附子汤证 (Cassia Twif and Radix Aconiti Lateralis Preparata plus Atractylodes Decoction Syndrome)桂枝附子去桂加白术汤证	Fangzheng
4	(No interior Syndrome)无里证 (Ephedra-Radix Aconiti Lateralis Preparata-Liquorice Decoction Syndrome)麻黄附子甘草汤证	Fangzheng
5	(Platycodon Grandiflorus Decoction Syndrome)桔梗汤证 (Liquorice Decoction Syndrome)甘草汤证	Fangzheng
6	(Bulbus Allii Fistulosi and Sus Scrofa Domestica Brisson Decoction Syndrome)白通加猪胆汁汤证 (Bulbus Allii Fistulosi Decoction Syndrome)白通汤证	Fangzheng
7	(heat pathogen)热 (curable)可治	Treatment methods
8	(Fructus Terminaliae Chebulae)诃黎勒 (porridge)粥 (Frctus Terminaliae Chebulae Powder)诃黎勒散	Seldom-used prescriptions
9	(Sores of immersion)浸淫疮 (Coptidis Rhizoma Powder)黄连粉	Seldom-used prescriptions

##### Path-Based Reasoning

A total of 1335 rules were mined, and 479 rules were selected through ontology reasoning. The comparison of the manual audit results and the model results showed an accuracy rate of 0.6124, a recall rate of 0.4906, and an *F*_1_ value of 0.5448. The observation revealed that path-based reasoning can extract a substantial number of potential rules; however, its accuracy is suboptimal. Using ontology reasoning for further screening not only enhances the *F*_1_ score but also alleviates the burden of manual review.

#### Implicit Knowledge Completion

A total of 107 triples were added based on transitivity and symmetry, and 14 contradictory triples were discovered and removed based on mutual exclusion. Ontology-based reasoning can effectively identify inaccurate knowledge in KG.

#### Tacit Knowledge Completion

Using association rule mining, a total of 27 rules with lift values greater than 1 were discovered. Of these, 21 were related to *Yang Ming Tai Yin Combined Syndrome*, 4 were related to *Yang Ming Syndrome,* and 2 were related to *Jue Yin Syndrome.* The *Yang Ming Tai Yin combined syndrome* exhibits the highest number of associated rules, which can be attributed to its prevalence in medical cases. Rules with higher repetition are easily mined by association rules.

All of the above rules were converted into triples and added to the graph.

### Quality Evaluation

KG after completion had higher density and lower sparsity, without contradictory rules. It was more consistent with prior knowledge and improved the representation results of graph representation models.

#### Completeness

**Figure 4:**
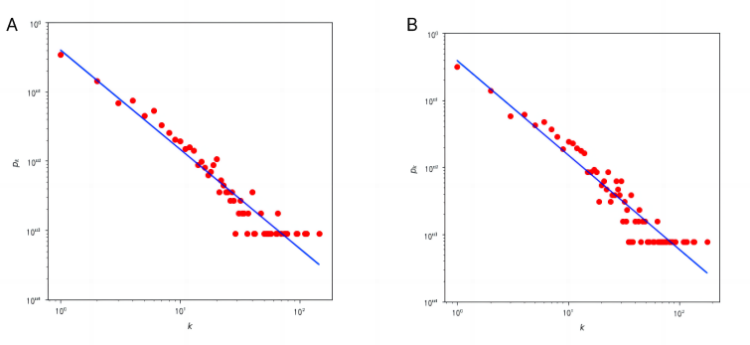
Distribution curves of the knowledge graph (A) before knowledge graph completion (KGC) and (B) after KGC.

The degree values both before and after KGC followed a power-law distribution (see Figure 4), indicating a core KG structure. The core syndromes before and after completion were both primary syndromes of *Six Channel Syndrome*, which is consistent with prior knowledge. Since the completion mainly focused on relations, the network density increased, and the sparseness decreased after completion (see Table 4).

**Table 4:** Description of the knowledge graph.

Time point	Node	Relation	Largest k	Average k	Network density	Slope of k distribution curve
Before KGC^a^	1255	4519	145	7.2016	0.0057	–1.4550
after KGC	1277	5162	179	8.0846	0.0063	–1.4058

^a^KGC: knowledge graph completion.

#### Accuracy

The number of contradictory rules was 14 before completion, and it decreased to 0 after completion.

Among the top 20 prescriptions ranked by CC after completion, core prescriptions accounted for 80% in the KG after completion, which is a 5% increase from the KG before completion. This indicates that the completion work made the graph more consistent with domain knowledge.

The proportion of symptoms related to *Exterior Syndrome* among the symptoms with the highest k-core values increased after KGC increased (see Table 5). These symptoms included aversion to cold, floating pulse, fever, spontaneous sweating, aversion to wind, body pain, tight pulse, headache, anhidrosis, cold limbs, and chest tightness. Given that *Six Channel Syndrome Differentiation* emphasizes the differentiation of *Exterior Syndrome*, the completed graph is closer to prior knowledge.

**Table 5:** The k-core value of symptom nodes before and after completion.

Time point	Largest k-core	Symptoms with the largest k-core	Proportion of symptoms related to exterior syndrome
Before graph completion	10	32	0.3438
After graph completion	12	43	0.3721

#### Usability

After completion, compared with before completion, the MR of each model decreased, the MRR was closer to 1, and Hits@N increased, suggesting that the representation performance of each model improved. Among them, the RotatE model changed the most (see Table 6).

**Table 6:** Knowledge graph embedding performance before and after completion.

Models at each time point		MRR^a^	MR^b^	H@1^c^	H@3^d^	H@10^e^
**Before completion**						
	TransE	0.2245	126.6173	0.1385	0.2502	0.3866
	RotatE	0.3682	125.0212	0.3077	0.3734	0.4878
	DistMult	0.2908	255.7703	0.2472	0.2991	0.3721
	ComplEx	0.3196	244.0631	0.2835	0.3189	0.3913
**After completion**						
	TransE	0.2414	114.0714	0.1507	0.2657	0.4256
	RotatE	0.3830	115.0664	0.3130	0.4009	0.5281
	DistMult	0.2944	233.3249	0.2512	0.3020	0.3731
	ComplEx	0.3265	231.3083	0.2875	0.3235	0.4081

^a^MRR: mean reciprocal ranking.

^b^MR: mean rank.

^c^H@1: hit rate of the first 1 tail entity predicted by the model.

^d^H@3: hit rate of the first 3 tail entities predicted by the model.

^e^H@10: hit rate of the first 10 tail entities predicted by the model.

## Discussion

### Principal Findings

We summarized the characteristics of TCM domain knowledge and designed a 3-step “path-ontology-entity” KGC plan. The plan can efficiently complete explicit knowledge, effectively reason about implicit knowledge, and mine tacit knowledge while maintaining good explainability. This paper explored the transfer of KG quality management systems to the TCM field and designed a comprehensive evaluation system for KGs in this field. The scheme was comprehensively evaluated from the 3 dimensions (completeness, accuracy, usability), each with its own set of quantitative indicators.

For the KG constructed around *syndrome*, core *syndrome* nodes that establish more connections with other nodes can offer additional information for syndrome differentiation. When there is a discrepancy between core symptoms or core prescriptions in prior knowledge and the KG, it can be inferred that the KG has not fully represented the knowledge, which can guide researchers in subsequently completing the relationships of specific categories. Nodes with a higher k-core value are key points connecting other nodes in the KG and often provide differential diagnostic information. The KG completed and evaluated using the aforementioned methods will provide accurate domain knowledge for tasks such as clinical decision support on syndrome differentiation and prescription recommendation.

We also explored KGE methods tailored for the TCM KG. Our model with RotatE achieved the best performance, followed by ComplEx, while TransE performed the least effectively. TransE was unable to handle complex relationships such as one-to-many, many-to-one, and many-to-many. RotatE more effectively captured directional relationships between entities and handled complex graph structures, which aligns better with the characteristics of KGs in the TCM domain. In ComplEx, entity and relationship embeddings no longer exist in the real space but in the complex space, capturing more information. This study can provide a reference for other intelligent diagnosis and treatment research with KG fusion.

### Comparison With Prior Work

Compared with existing research, this study analyzed the characteristics of TCM domain knowledge and proposed a methodological theory for KGC in the TCM domain, which enhances the systematic and comprehensive nature of the completion process. In addition, outlier detection is a completion method not used in existing studies. In terms of improving KG accuracy, this method can effectively identify missing knowledge in KGs, while ontology-based reasoning is more appropriate for identifying inconsistent knowledge. These 2 methods complement each other. Although complex networks have been used to mine knowledge within KGs [55], there is a lack of literature on their use for graph quality assessment. The KGC and evaluation approach proposed for the TCM domain in this study may serve as a reference for KG construction in this field.

### Limitations of the Study

The KG constructed in this study is of a small scale. A thorough validation of the proposed methods is necessary when applied to larger or more diverse data sets. The methods used in this paper do not occupy a lot of computational resources. However, the use of the random walk approach may involve higher time complexity than dynamic programming or heuristic search algorithms. Association rule mining extracted only a small amount of tacit knowledge, which may be related to the number and representativeness of medical records. In the medical cases, 45% of the syndromes were Jue Yin Syndrome, and 35% were YangMing-TaiYin Syndrome. The insufficient data quantity for other syndromes resulted in low lift values in the association rule mining and prevented the discovery of “Syndrome-Symptom” associations. In subsequent studies, we plan to increase the number of medical records and explore other rule mining methods. For instance, generative adversarial networks can be used for data enhancement, thereby making the sample distribution more balanced.

### Conclusions

The lack of KGC and evaluation methodology restricts the development of KGs in the TCM field. This study first analyzed the knowledge levels within the TCM domain and proposed a 3-step completion plan of “path-ontology-entity” based on the characteristics of each knowledge level: Path reasoning was used to mine explicit knowledge, ontology reasoning was used to mine implicit knowledge, and association rule analysis was used to mine tacit knowledge. An evaluation system including 3 dimensions—completeness, accuracy, and usability—was designed, with each dimension using quantitative evaluation indicators to assess the quality of the completed KG. The results indicate that, under the guidance of the proposed methodology, the completed graph resulted in improvements across all dimensions. In terms of completeness, 22 nodes and 643 edges were added to the completed graph, and the network density was increased. In terms of accuracy, the core prescriptions among the top 20 CC prescriptions of KG after completion increased by 5% compared with those before completion, and the proportion of symptoms related to syndromes with the highest k-core value increased, suggesting that KG after completion is more in line with prior knowledge. In terms of usability, in the triplet prediction task, the completed KG enhanced the performance of all graph representation models. The “path-ontology-entity” 3-step completion plan effectively improved the integrity, accuracy, and availability of KGs, and the 3-dimensional evaluation system provided a comprehensive assessment of KGC. In addition, the RotatE model outperformed other commonly used models in the graph representation of KGs within the TCM domain. Our study provides a methodological reference for the completion and evaluation of TCM KGs.

## Abbreviations

CC: closeness centrality
GFO: General Formal Ontology
KG: knowledge graph
KGC: knowledge graph completion
KGE: knowledge graph embedding
MR: mean rank
MRR: mean reciprocal ranking
PRA: path ranking algorithm
TCM: traditional Chinese medicine
TCMLS: Traditional Chinese Medicine Language System
